# Complications Associated With Inferior Vena Cava Filter Retrieval: A Systematic Review

**DOI:** 10.7759/cureus.55052

**Published:** 2024-02-27

**Authors:** Amanda K Rodriguez, Anjali Goel, Vasavi R Gorantla

**Affiliations:** 1 Internal Medicine, St. George's University School of Medicine, St. George's, GRD; 2 Biomedical Sciences, West Virginia School of Osteopathic Medicine, Lewisburg, USA

**Keywords:** inferior vena cava filters, deep vein thrombosis, pulmonary embolism, inferior vena cava filter retrieval, inferior vena cava

## Abstract

Inferior vena cava (IVC) filters have been used successfully in high-risk patients to prevent thromboembolism. The filters are widely created as retrievable devices, but complication rates progressively increase during IVC filter retrieval. This study aims to analyze IVC filter retrieval cases and associated complications during and following the procedures regarding dwell times, specific filter types, filter positioning, and advanced retrieval techniques. This review followed the Preferred Reporting Items for Systematic Reviews and Meta-Analyses (PRISMA) guidelines to select and analyze relevant articles. A literature search for articles was performed on September 23, 2023, through three research databases: PubMed, ProQuest, and ScienceDirect. The keywords used to identify relevant publications were “IVC Filter retrieval AND complications” and “IVC filter removal AND complications”. The articles before 2012 were excluded. Relevant articles were selected based on the inclusion and exclusion criteria. In total, 20,435 articles were found: 812 from PubMed, 15,635 from ProQuest, and 3,988 from Science Direct. Among the exclusions were 18,462 articles, which were excluded in the automatic screening process, leaving 1,973 for manual screening. The manual screening of articles was conducted based on title, abstract, article type, duplicates, and case reports, where 1,918 articles were excluded. Ultimately, 55 articles were included in this review. This study demonstrates that IVC filter retrievals have significant complication rates. Many complications have a common theme: prolonged dwell time and lost follow-up appointments. Therefore, importance should be placed on patient education and implementing strict protocols regarding the timelines of IVC filter removals.

## Introduction and background

Extensive research has been conducted on the inferior vena cava (IVC) filtration device since the 1960s. This intervention has proven to be beneficial for patients who are at a heightened risk of developing pulmonary embolism (PE) or deep vein thrombosis (DVT) [1,2]. Patients with specific indications, such as a history of PE in the past, protracted immobility, or persistent risk factors (e.g., patients who are unable to take blood thinners due to an increased risk of intracranial hemorrhage, hemorrhagic stroke, or gastrointestinal bleeding), are also candidates for IVC filters [3].

IVC filters are basket-like filters designed to capture venous emboli that discharge from the lower extremities and form below the filter. This prevents the emboli from reaching the lungs, where they could otherwise cause clinically significant PE [1,4]. Following the approval of IVC filters by the United States Federal Drug Administration (FDA) in the early 2000s, their use increased. Rates of IVC filter implantation have increased annually. In cases where initial anticoagulation treatments for patients with acute DVT are contraindicated or have proven to be ineffective, IVC filters have been suggested as an alternative [5,6].

Nevertheless, caval occlusions were documented in 10% of permanent filters at the one-year follow-up and increased to 30% by the nine-year follow-up [7]. Thrombosis at the venous access site, migration of filters, and penetration of vessel walls are all considered serious complications [1,8-10]. The long-term complications associated with persistent filters have prompted the development of retrievable filters, which aim to preserve the benefits of filtration while mitigating these issues [2,11]. The pursuit of developing a retrieval method that is both secure and efficient has intensified in response to the escalating rate of filter implantation since 2003 [12]. The potential complication rates associated with temporary IVC filters were acknowledged by the US FDA in 2010. The agency advised that the filters be removed when the elevated risk of PE is no longer present and after a risk-benefit analysis for removal per patient had been completed [13,14]. With retrievable IVC filters, complications such as small bowel perforation, abdominal complaints, and concurrent aortic and vertebral penetration have been reported during and after the procedures [15]. Atrial arrhythmia and cardiac tamponade are complications that may arise from embolization of a filter fracture [16]. The objective of this review is to examine and evaluate ongoing scholarly works in order to draw attention to the documented complications that are linked to transient IVC filter removal methods.

## Review

Methods

The Preferred Reporting Items for Systematic Reviews and Meta-Analyses (PRISMA) guidelines were followed to perform this systematic review. The search for articles was conducted on September 23, 2023, through the three research databases: PubMed, ProQuest, and ScienceDirect. The query used in all of the search databases includes: “IVC Filter retrieval” AND “complications,” “IVC filter removal” AND “complications.” Articles not written in English, duplicate articles, and articles published before 2012 were excluded during the screening process. Articles were screened based on title, abstract, and study type during the manual screening process. Our initial search from the databases resulted in 11,155 articles. We screened the selected articles according to the inclusion and exclusion criteria, and 55 were yielded.

Inclusion Criteria

The inclusion criteria consisted of studies written in English, studies conducted on humans, studies relevant to our topic and research question, and peer-reviewed full-text articles, including clinical trials and meta-analyses.

Exclusion Criteria

**Figure 1:**
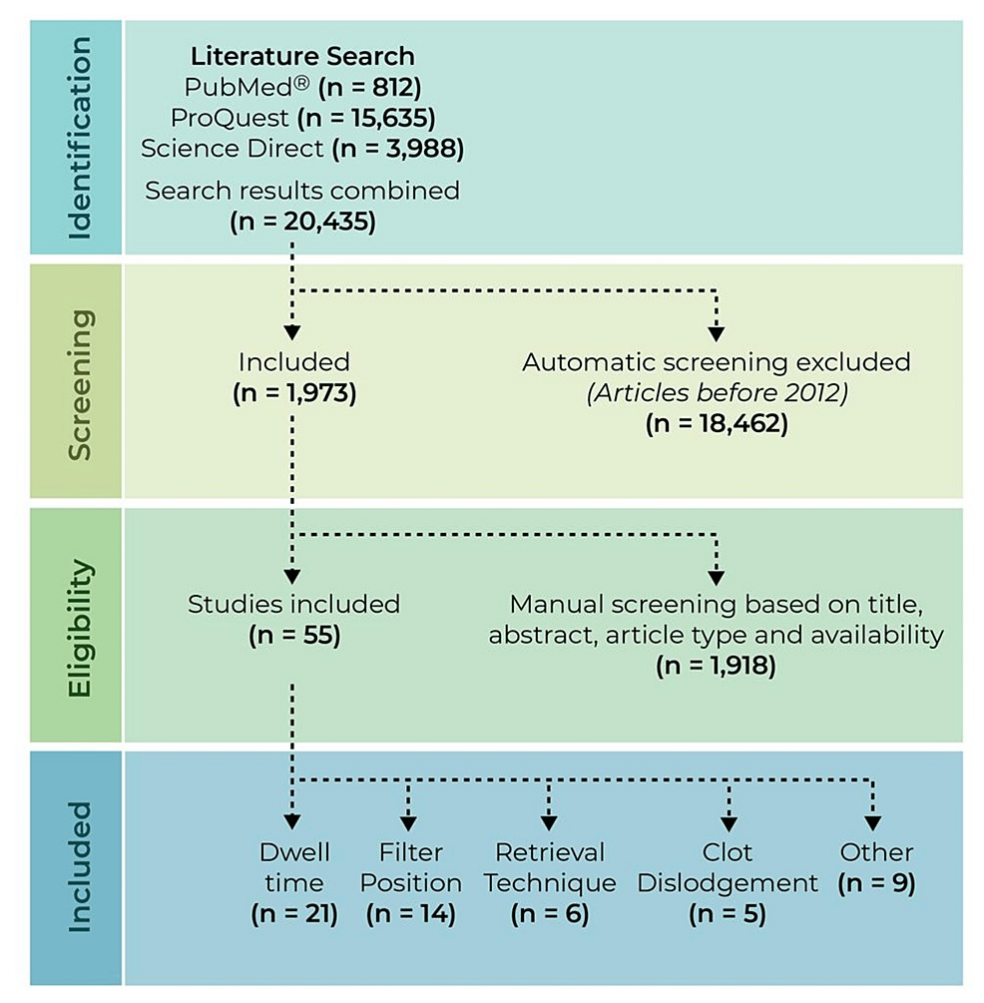
PRISMA flowchart describing the literature screening of studies concerning the complication of IVC filter retrieval PRISMA flowchart describing the literature screening of studies concerning the complication of IVC filter retrieval [17]. PRISMA: Preferred Reporting Items for Systematic Reviews and Meta-Analyses; IVC: inferior vena cava The PRISMA statement guidelines were followed to perform the screening for this literature review [17].

Exclusion criteria included duplicates of articles, case reports, animal studies, narratives, systematic reviews, and manual reviewing. Figure 1 illustrates the inclusion and exclusion process.

Results 

Three databases, including PubMed, ProQuest, and ScienceDirect, were investigated for this systematic literature review. Our initial research resulted in 20,435 articles being found: 812 from PubMed, 15,635 from ProQuest, and 3,988 from Science Direct. Using database filters, our automatic inclusion of articles consisted of peer-reviewed full-text articles, meta-analyses, clinical trials, randomized control trials, and those published between 2012-2023, which removed 18,462 articles. This left 1,973 articles for manual screening based on title, abstract, article type, duplicates, and case reports, where 1,918 articles were excluded. Two co-authors reviewed the articles for content relevance which resulted in a final inclusion of 55 articles. Of these included articles, we reviewed 42 retrospective studies, eight prospective studies, two observational reviews, two questionnaire-based studies, one randomized-control trial, and one clinical study. 

In the review of these articles, studies were carefully chosen by the co-authors with significant sample sizes, which increased the power of the results. To minimize bias, studies that took place at one institution were chosen to reduce the risk of confounding results among the population studied. To further investigate the complications of IVC retrieval, a variety of filter brands, surgical removal procedures, and dwell times were studied to identify the risks and benefits. Upon exploring the articles, we were able to identify relationships contributing to IVC filter removal complications. We further categorized these findings in our discussion as filter position, dwell time, retrieval technique, and clotting. Changes in these variables play an important role in the success or failure of filter retrieval. Our hopes for this review are to establish patterns for complications that can serve as a guide for future IVC filter retrievals to minimize the complications among patients. All included studies, number of cases, significant findings, and conclusions are summarized in Table 1.

**Table 1:** Articles focusing on the complications of IVC filter retrieval IVC: Inferior vena cava; DVT: deep vein thrombosis; PE: pulmonary embolism; CT: computed tomography; VTE: venous thromboembolism; GT: Gunther Tulip; SOGL: snare-over-guide wire loop; INR - international normalized ratio; LMWH: low-molecular-weight heparin; IVCF: inferior vena cava filter; CI: confidence interval; PR: pulse rate; GTF: Gunther Tulip filter ; INR: International normalized ratio; LMWH: low-molecular-weight heparin; GI: gastrointestinal ; FDA: Food and Drug Administration ; IVCF: inferior vena cava filter; HA: hook/apex; SR: standard retrieval; AER: advanced endovascular retrieval; MTGL: modified guidewire loop; MDST: multidisciplinary surveillance team

S.No	Author	Country	Design & Study Population		Findings	Conclusion
1	Johnson et al., 2010 [1]	USA	Prospective study (n=100)		The technical success (defined as the deployment of the filter such that it was judged suitable for mechanical protection from PE) of 100% was achieved in all subjects. Of all the tested subjects, eight cases resulted due to recurrent PE, three cases of symptomatic occlusion/thrombosis, and two cases of filter migration were reported in the study. There was no damage observed due to filter embolization or the filter fracture. Moreover, the clinical success of IVC retrieval was achieved in 92.3% (36 of 39 subjects) upon the mean retrieval period of 67.1 days of implantation. In addition, the study suggested that all deaths (n= 17) and deep vein thrombosis (n=18) were results of pre-existing or intercurrent illnesses and not related to the filter device. Lastly, the deaths were concluded to be unrelated to PE.	The Option IVC filter retrieval and placement were achieved safely with high clinical and technical success rates.
2	Pellerin et al., 2013 [2]	France	Prospective study (n=503)		From all of the 503 patients, ALN IVC filters were placed, only 29 patients were followed in this study as those were the only ones who had their filters for at least one year following implantation. No history of post-thrombotic syndrome, DVT, or PE was reported in their patients since the filter implantation. The mean interval between the time of the implantation, and the time of retrieval was 25.6 months. However, for eight of these patients, the filter extraction was upon a mean delay of 32.6 months as they had received the filter at least two years earlier. The longest dwell time of the IVC filter was 40 months. All of the filter extraction was successful without complication despite the filter's showing 15 degrees of tile in 11 patients and more than 15 degrees in two patients.	Optional ALN IVC filter retrieval can be achieved with safety and feasibility for the longest dwell time of more than one year upon the implantation.
3	Tapson et al., 2017 [3]	USA	Prospective study (n=163)		The study showed the primary efficacy endpoint in all 163 patients (100%) (95% CI, 97.8%–100%, P<0.01) which was to achieve freedom from the clinically significant pulmonary embolism or fatal pulmonary embolism 72 hours after filter removal. Upon the removal of the filter, the recurrence of the new or worsening acute proximal DVT was found to have occurred in the first seven days in a time-dependent manner in 11 (7%) patients. There were no (0%) catheter-related infections found in the bloodstream. Five major bleeding events occurred (3.1%), and a significant thrombus in the IVC filter occurred in 14 (8.6%) patients. Moreover, upon the admission of patients to the ICU prophylactic anticoagulation was not administered for a mean of 5.5 days.	This trial concluded that the novel device of combining the IVC filter and the central venous catheter can help prevent clinically significant and fatal pulmonary embolism among critically ill patients as it can be placed safely at the bedside without fluoroscopy with low risk of complications.
4	Jaberi et al., 2020 [4]	Canada	Retrospective study (n=1123)		Identified a cohort with unretrieved IVC filters inserted between January 2001 and December 2013. These patients were invited back to the clinic for review with CT imaging to determine complications, if any, and offer removal. Data collected included demographics, complications and retrieval characteristics. 289 patients were discovered to still have a filter in situ. Of these, 193 patients were deceased. 89 patients were notified, with no current contact information available for the remaining seven. 36 attended for review, 20 females and 16 males, with an average age of 63.5 years. Complications identified at CT were two occluded IVCs (5.8%), four fractured filters (11.7%), and filter penetration in all cases (37.5% grade 2, 56.25% grade 3). 16 patients agreed to proceed with filter removal, 10 declined the opportunity and 6 were unfit or had ongoing indications for the filter. Two are awaiting removal and two had IVC occlusion. Subsequent retrieval was successful in 93% of cases (14/15). The mean time to removal from the implant was 3,846.9 days (SD 980.3). Advanced techniques were utilized in 10 cases and there were no mortalities or morbidities.	Retrievable IVC filters are not benign and practitioners need to be aware of regulatory guidelines. Unretrieved filters can be successfully retrieved using standard and advanced methods with low morbidity and mortality.
5	Stavropoulos et al., 2016 [5]	USA	Prospective study (n=200)		The Denali IVC filters showed promising results in filter placement with a technical success rate of 99.5% (199 patients). Filters also showed 95% of clinical success where the PR rate was only 3% with 5 patients having small subsegmental PE and 1 patient having lobar PE. However, the DVT was found to be worsening in about 26 patients (13%). Further, the study showed the filter retrieval technical success rates of 97.6% (n=121) after a mean dwell time of 200.8 days. During this mean retrieval time period, no complications related to filter fracture, migration, or tilt greater than 15 degrees at the time of filter retrieval or follow-up were observed.	The study concluded that the Denali IVC filter placement and retrieval showed a high success rate while maintaining a low complication rate in the clinical trial.
6	Wassef et al., 2017 [6]	Canada	Retrospective study (n=464)		Acute deep vein thrombosis with a contraindication to anticoagulation was the indication for 206 (44.4%) filter insertions. No contraindication to anticoagulation could be identified in 20.7% of filter placements. 30.6% were placed in those with active cancer, in which mortality was significantly higher. Only 38.9% of retrievable filters were successfully retrieved. Recent surgery increased the likelihood of successful filter retrieval while a decreased likelihood of filter retrieval was associated with increasing age, active cancer, lung disease requiring oxygen, prior venous thromboembolism (VTE), either initial deep vein thrombosis (DVT) alone or the combination of DVT and pulmonary embolism (PE), major bleeding or high risk of bleeding.	Inferior vena cava filters were placed frequently in patients with weak or no guideline-supported indications for filter placement and in up to 20% of patients with no contraindication to anticoagulation. The high rates of cancer and the high mortality rate of the cohort raise the possibility that some filters are placed inappropriately in end-of-life settings. The rate of successful filter retrieval was low.
7	Given et al., 2008 [7]	USA	Retrospective study (n = 322)		The GT IVC filters were inserted for a variety of reasons, one of them being prophylaxis of PE in perioperative patients. 205 attempts were carried out in the study of which 15 failed. The retrieval rate of the GT IVC filter was found to be 92% after a dwell time of 76.95 days. The study showed three minor complications associated with the insertion of the GT filter and five related to retrieval. It also showed that the GT IVC filter's longer dwell time (76.40 days) before its retrieval is safe compared to what was suggested by the manufacturer at or before 14 days to avoid the risk of endothelialization.	The retrieval periods of the GTF have continued to increase besides the initial recommendation of 14 days. It was also concluded that increased dwell times may complicate the successful retrieval of the IVC filter.
8	Kuo et al., 2011 [8]	USA	Retrospective study (n=10)		The complex retrievals were assessed in 10 patients who had indicated filters due to venous thromboembolism (VTE) and contraindication to anticoagulant, VTE and inability to maintain anticoagulation therapy, pulmonary embolism while undergoing deep vein thrombosis, and a patient with a massive pulmonary embolism. Complex retrieval techniques were successful in 100% of procedures that had embedded tips. Advanced techniques included snare-over-guide-wire loop (SOGL) modified using a 10F Flexor sheath or 14 F bright-tip sheath, rigid endobronchial crocodile jaw forceps, SOGL with additional flexible endoscopic alligator jaw forceps, coaxial bi-sheath and bi-snare method, laser-assisted sheath technique with photothermal ablation, and SOGL with laser-assisted sheath technique. In conjunction with the aforementioned removal techniques, IVC recanalization, venoplasty, and/or thrombolysis were deployed when needed. Histological tissue analysis revealed all 10 patients to have neointimal hyperplasia, dense fibrosis, and scant native intimal tissue.	The study found significant successful findings in using complex techniques for embedded optional and permanent IVC filters. The results are significant because the mentioned filters were previously considered non-retrievable. The study found chronic filters to have neointimal hyperplasia and dense fibrosis where the filter was attached. Excimer laser technology was used to ablate the abnormal tissue during retrieval and provides a promising advancement in chronic IVC filter removal success.
9	Ballard et al., 2016 [9]	USA	Retrospective study (n=26)		There were 26 attempts at retrieval, at a mean of 42.9 days from placement. There were nine failures (34.6%) due to the inability to snare the hook, the non-collapsible IVC filter, and the presence of procedural pain. The study found that the median dwelling time for retrieved filters was 31 days and 53 days for failed retrievals (p= .0483). 92% (24/26) of the attempts were accessed through the right common femoral. vein. In the remaining two, access was through the left common femoral vein and right internal jugular vein, both of which were unsuccessful. IVC filters in alignment with the cephalocaudal axis were retrieved in 13/16 cases, and tilted IVC filters were retrieved in 4/10 cases (40%; p=.0461).	The study found that the main causes for the failure of retrieval were due to filter duration and alignment. Longer IVC filter duration times and misalignment with the IVC filter lumen axis were related to more retrieval failures. The study findings place importance on removing filters as soon as medical necessity is no longer indicated. The study also mentions that there was a low rate of patient follow-up and suggests future IVC placement to have stricter patient follow-up protocol.
10	Iliescu et al., 2011 [10]	USA	Observational study (n=62)		In the case of complications arising during retrieval, additional information can be found by CT, cone beam angiographic CT, and rotational angiography. CT proved to be successful in identifying caval wall penetration in 85.9% of 64 filter retrievals. Adjunctive techniques were used when the IVC filter hook could not be engaged using traditional snares. Loop snare with single access and tip-deflecting wire was used when the filter was in misalignment with the longitudinal axis. When a tilted filter apex or hook couldn't be engaged, a single access stiff wire-displacement technique could be used. For cone or umbrella-shaped IVC filters a jugular approach is common and for trapezoid-shaped filters, a femoral approach is commonly used. When single-access approaches fail in displacing a filter, dual access wire and snare technique can be deployed which is described as "flossing". When those approaches fail, the balloon-displacement technique can be used to separate the filter from the caval wall using an interposed angioplasty balloon. This was reportedly successful in 8/10 severely tilted filters. The single-access sling technique using a wire loop-and-snare technique can also be used for an embedded hook, which was reported successful in the removal of a filter tilt of 90 degrees. Rigid bronchoscopy forceps, which are more invasive, are used for dissection of severely embedded IVC filters. Migration of IVC filters can occur frequently, but cardiac/pulmonary migration can be lethal usually requiring a surgical approach.	The study concludes that the majority of temporary IVC filters are not temporary at all. They also state that the majority of retrievals are uneventful, but the techniques described prove effective when the need arises. An important suggestion for the establishment of methodical follow-up is notable to increase retrieval rates, as well as tertiary and quaternary referral systems.
11	Hoppe et al., 2007 [11]	USA	Retrospective study (n=110)		Therapeutically anticoagulated patients (Group 1) included 65 attempted filter retrievals in 61 patients by the measured INR or dosing when receiving LMWH. Four retrievals were not successful. In patients receiving oral anticoagulation, the median INR was 2.35 (range=2-8). Prophylactically anticoagulated patients (Group 2) comprised 23 successful filter retrievals in 22 patients receiving LMWH. Not therapeutically anticoagulated patients (Group 3) included 27 attempted filter retrievals in 27 patients. Six retrievals were not successful. Five patients were receiving oral anticoagulation with a subtherapeutic INR (median=1.49; range=1.16-1.69). No anticoagulation medication was administered in 22 patients. In none of the groups were hemorrhagic complications related to the retrieval procedures identified.	Retrieval of vena cava filters in anticoagulated patients is safe. No indication of interruption or reversal of anticoagulation for the IVC filter retrieval.
12	Kuo et al., 2009 [12]	USA	Retrospective study (n=59)		Three IVC occlusions necessitated recanalization to facilitate retrieval. High-risk retrieval with the use of various techniques with aggressive force was successful in all 13 patients (100%). Partial caval thrombosis occurred in the first four patients (31%) but did not occur after procedural modifications were implemented. There were no complications at clinical follow-up (mean: 221 days; range: 84–452 days).	Using modified retrieval techniques combined with adjunctive endovascular maneuvers, removal of adherent IVC filters implanted for up to five years was feasible. Despite the use of aggressive techniques, the only major complication encountered in this small series was caval thrombosis and protocol modifications appeared to subsequently reduce this risk.
13	Kleedehn et al., 2018 [13]	USA	Retrospective study (n=187)		Complex retrieval and increased fluoroscopy time were associated with hook apposition (p<0.01). A larger tilt angle at placement was not associated with the need for a complex removal technique (p=.22). Complex retrieval techniques were associated with longer dwelling times (p=0.02). Filter type, gender, and age were not associated with complex retrieval techniques (p=0.58, p=0.90, p=0.99, respectively). Neither hook direction nor relationship to the tilt was related to the need for a complex retrieval technique or increased fluoroscopy time (p=0.25, 0.23, p=0.18, 0.23).	The study found evidence against previous studies that suggested the direction of the hook-tilt relationship as a factor for increased fluoroscopy time and the need for complex retrieval techniques. Complex retrievals were concluded to be associated with increased filter placement angle correlated to a larger angle at removal and hook-wall apposition.
14	Quencer et al., 2020 [14]	USA	Observational study (n=228)		Advanced techniques were found to increase the overall complication rate four-fold and a 13x increase in major complications. Advanced technique alone resulted in 5.4x greater fluoroscopy time and 3.6x greater radiation time. In the case of filter embedding, rigid bronchoscopy forceps were used. Major complications included symptomatic IVC pseudoaneurysm requiring balloon tamponade. Minor complications included two filter leg fractures with embolization to the pulmonary artery, which were snared in time, and an asymptomatic IVC pseudoaneurysm. Procedural complications included leg fractures with embolization and arterio-venous fistulas present after the procedure. The sling technique has resulted in a 20% overall complication rate, specifically leg fracture and IVC dissection. Excimer laser technique complications included caval injuries (7%), venous pseudoaneurysm (4%), and contrast extravasation (3%).	The study concludes complex filter retrievals provide technical difficulty, as well as patient morbidity. The study suggests in the removal of difficult filters using advanced techniques, that tamponade balloons and stent-grafts are readily available during the procedure.
15	Genovese et al., 2015 [15]	USA	Retrospective Study (n=9)		Nine patients had symptomatic GI complications associated with a retrievable IVC filter (two G2 Recovery, seven Celect; six women; age range, 17- 81 years). All patients had small bowel perforation on a computed tomography scan, and four were confirmed by esophagogastroduodenoscopy. Concomitant aortic and vertebral penetration occurred in seven and five patients, respectively. Patients presented with various abdominal complaints; one patient presented with acute sepsis. Two patients underwent laparotomy without complications. The remaining seven patients had attempted endovascular retrieval, six of which were successful. One patient’s IVC filter was unable to be retrieved, and he was managed medically. Of the six patients who had successful endovascular retrieval, all had resolution of their symptoms and no complications, one patient had transient sepsis because they were not receiving periprocedural antibiotics. A follow-up CT scan was performed 48 to 72 hours after endovascular retrieval and duodenal leak was ruled out in all the patients. Long-term follow-up demonstrated continued resolution of GI symptoms without further episodes of deep venous thrombosis or pulmonary embolism.	GI complications of retrievable IVC filters have a wide spectrum of symptoms and frequent concomitant aortic and vertebral penetration. Endovascular retrieval can be safely used as a first-line therapy even in small bowel and aortic penetration.
16	Vijay et al., 2012 [16]	USA	Retrospective study (n=548)		A total of 63 fractured recovery, G2, and G2 Express IVC filters were identified, overall fracture rate=12%. The fracture rate for only filter arms and/or legs was 6%. The fracture incidence increased with longer filter dwell times. Success rates for removal of the non-fractured component (main body) and fractured components (arm or leg) were 98.4% and 53.4%, respectively. The distal embolization rate of fractured filter components was 13%. No immediate clinically significant complications associated with fracture component embolization or filter removal were present. One patient was encountered with symptoms related to their fractured filter.	IVC filter fracture rates increase with longer dwell times. Removal of fractured filters and their components (arms and legs) can be achieved effectively and safely. Clinically significant complications of IVC filter fracture are rare, and there were no immediate clinical sequelae related to the embolization of fracture components.
17	Kai et al., 2006 [18]	Japan	Questionnaire-based study (n=50)		The study divided the 50 patients into two groups where Group A had 18 patients who received permanent IVC filters and 32 patients in Group B received temporary IVC filters. as their primary treatments. It was reported that there were no major complications found in either of the groups during IVC implantation, however, the mortality rate after implantation was found to be higher in Group A (35%) than in Group B (16%). Moreover, pulmonary thromboembolism was found to have recurred and caused death in Group A patients in 18% but none of the Group B patients showed any recurrence as they received temporary IVC filters. Lastly, the study reports that 14 Group B patients were not given permanent IVC filters upon the removal of the temporary IVC filters, all of whom survived.	The study concluded that the temporary IVC filters can be used safely and are highly efficacious in preventing the recurrence of pulmonary thromboembolism. Moreover, the prognosis of the temporary IVC filters is remarkable.
18	Hansmann et al., 2019 [19]	USA	Retrospective study (n=204)		The majority of filters were placed for venous thromboembolism (200/204, 98%). Of 204 filters, 38(19%) were retrieved at a median 186 days post-placement (range 3–665 days), 112(55%) converted to permanent devices, 44(22%) patients were deceased, and 10(5%) patients were lost to follow-up after transfer to an outside healthcare facility. Filter removal was less likely in patients with subarachnoid hemorrhage (18% vs. 35%) and malignancy (5% vs. 25%). Filter type, gender, neurosurgical procedure, and insurance status did not demonstrate a significant association with filter retrieval.	IVC filter retrieval rates in neurosurgical patients are low. Those neurosurgical patients with intracranial hemorrhage or malignancy requiring IVC filters have a lower likelihood of filter retrieval and may benefit from the use of permanent devices.
19	Tao et al., 2016 [20]	Canada	Retrospective Study (n=810)		During the study period, 1123 IVC filter procedures were performed; 69% (n=810) were insertions and 31% (n=313) were retrievals. Patients receiving filters had an average age of 61.4 years, and 53.3% were male. 408 filters (51.5%) were placed with absolute indications, 214 (27.0%) for relative indications, 138 (17.4%) prophylactically, and 32 (4.0%) for reasons outside the established guidelines. 663 were retrievable filters and the successful removal rate was 41.6% (n=276); the mean time to the first retrieval attempt was 76.4 days. Significant predictors of filter removal were thrombosis follow-up and the ordering service since filters ordered by medical specialties were less likely to be retrieved than filters ordered by surgical specialties. Compared with discharge summaries without filter management instructions, those with plans had higher filter retrieval rates. Filter-related complications were observed in 57 patients and an additional 44 patients had long-term complications associated with filter removal. Thrombosis formation at the filter site was the most commonly observed complication (n=27), followed by filter embedment (n=10). Evidence suggests complication rates for retrievable filters to be higher than for permanent filters because of the increasing number of retrievable IVC filters being inserted combined with the lack of retrieval.	Complications relating to long indwelling times and recent FDA guidelines necessitate a multidisciplinary and systematic follow-up protocol implementation to optimize filter retrieval rates and to ensure high quality of care.
20	Tse et al., 2017 [21]	United Kingdom	Retrospective study (n=393)		A total of 393 retrievable IVC filters were placed, with 254 of them with an indication of pre-operative PE prophylaxis. Of the 254 IVC filters inserted, attempts to remove were 168/254 (66.1%), and there was a mean dwell time of 59.5 days. On the first attempt, 143 filters were successfully removed (85.1%) and 25 cases failed or had to abort due to filter tilts and thrombus (14.9%). Seven cases were successfully removed on the second attempt, and they were failed or aborted notably due to the presence of filter thrombus. No attempt at retrieval was made in 86 patients (33.9%) because a significant proportion of them were surgical cancer patients (p<0.0107). The relationship was significant between cancer surgery and shorter survival time (p<0.0001). Overall, 150 filters were removed (59%) and (58%) of those were removed in the first three months.	The study did not note any significant findings in filter retrieval failure due to increased dwelling time. The study concluded that preoperatively inserted filters for non-cancer surgical patients had a high retrieval rate. They noted importantly that the excellent retrieval rate was in conjunction with a plan to retrieve the filter at the time of placement, and close follow-up was maintained. A large portion of non-retrieved IVC filters were due to surgical cancer patients who died with the filter still in place.
21	Charles et al., 2009 [22]	USA	Retrospective analysis (n=140)		27 attempts were made in the G2 IVC filter removal after a mean period of 122 days in 26 patients. All of the filter retrieval procedures were successful with a 100% technical success rate. Five cases (18.5%) showed tilting of the filter (≥15 degrees). However, one of the cases showed the filter to be embedded into the right lateral wall of the IVC. Using this filter, the study showed none of the other complications of retrieval such as filter thrombosis, significant filter migration, filter fracture, and caval occlusion.	G2 IVC filter retrieval shows a high technical success rate with limited impact by filter tilting within this small study group. Moreover, these filters show a low complication rate and low rate of thrombosis, symptomatic PE, and a good retrieval safety profile.
22	Kuo et al., 2020 [23]	USA	Prospective study (n= 500)		The 8.5 years dwell time period of various IVC filters was followed in this study as the 3x standard retrieval force technique had failed and the laser sheath powered by a 308-nm XeCl excimer laser system was used. This new technique showed promising results with 99.4% (497/500) of cases showing successful laser-assisted retrieval with a mean filter dwell time of 1,528 days. This technique showed a retrieval of 414 retrievable type filters and 86 permanent type filters. Moreover, the average amount of force used during laser-assisted retrieval was 3.6 lbs whereas during the failure attempts without the laser or standard techniques was 6.4 lbs. This technique also showed a complication rate of only 2.0% (10/500) being <5% significantly and all of the patients were successfully treated. Lastly, the study showed that successful retrieval of these filters allowed cessation of the anticoagulation in 98.7& of the patients and helped to alleviate filter-related morbidity in 98.5% of cases.	The excimer laser sheath technique is safe and effective for removing stubborn embedded IVC filters without the use of high-force retrieval attempts. Moreover, this percutaneous technique has allowed avoiding the use of invasive open surgery procedures when this technique can be used safely to remove a variety of IVC filters regardless of their dwell time, using minimal force.
23	Aurshina et al., 2019 [24]	USA	Retrospective study (n=604)		Retrieval rate=30%. A telephone survey was conducted for 42 patients with retained filters who were identified as possible candidates for retrieval. There was no difference between the men and women in terms of demographics and comorbidities. The survey showed that 12% of patients were not aware of having an IVC filter, and 23% knew that it could be removed. Women were significantly more likely than men to know the risks and benefits of IVC filter placement (42.8% vs 14.2%), but no significant difference in knowledge of the long-term complications of indwelling filters. The majority of patients (88%) had an established relationship with a primary care provider, 21.4% followed up with the team of physicians of the hospitalization for IVC filter placement. Better education about IVC filters would have improved follow-up in the opinion of 97.6% of patients. 50% relocated since filter placement and 35.7% changed their telephone number. There was no difference regarding the use of the Internet and interest in receiving educational material, but women (42.8%) significantly preferred receiving health-related communication by electronic mail, whereas men (64%) preferred telephone calls. The majority of patients (59.5%) had watched commercials for IVC filter lawsuits, among whom 26% sought discussion with a medical provider after watching the commercial. The main cause for no follow-up was “unaware of risks of leaving the filter” (69%).	Vascular specialists should educate the patient and family about IVC filters as well as long-term effects to optimize the patient’s compliance. Electronic communication for follow-up may help capture patients who relocate and change phone numbers and seems to be of particular interest to women compared to men.
24	Kallini et al., 2021 [25]	USA	Retrospective study (n=599)		A total of 599 patients (273 women and 326 men) had undergone successful IVCF placement. Preoperatively, 232 patients had been scheduled for IVCF removal within 3 months after placement. 367 patients had not been scheduled for removal. The indications for placement included the failure of anticoagulation, a contraindication to anticoagulation (eg, bleeding), preoperative prophylaxis, and others. Of the 232 patients scheduled for IVCF removal during preoperative consent for IVCF placement, 103 (44%) had undergone successful IVCF removal (mean interval from placement, 107 ± 100 days). Of the 367 non-scheduled patients, 89 (24%) had undergone successful IVCF removal (mean time, 184 ± 215 days). We found a significant improvement in the IVCF removal rate between the scheduled and non-scheduled patients. Three patients (all from the scheduled group) had a clot burden within the IVCF, which meant they were inappropriate for removal. These patients were rescheduled and had eventually undergone uncomplicated removal.	Scheduling IVCF removal during the placement encounter significantly increased the IVCF removal rate. A viable option for institutions where clinic time/resources are limited/unavailable and for patients who face difficulty in traveling for clinical evaluations.
25	Al-Hakim et al., 2014 [26]	USA	Retrospective study (n=217)		Filter retrieval was attempted 231 times in 217 patients (39% female, 61% male; mean age, 50.7 y), with success rates of 73.2% (169 of 231) and 94.7% (54 of 57) for routine and advanced filter retrieval techniques, respectively. The overall filter retrieval complication rate was 1.7% (four of 231); complications in four patients (with multiple complications in some cases) included IVC dissection, IVC intussusception, IVC thrombus/stenosis, filter fracture with embedded strut, IVC injury with hemorrhage, and vascular injury from complicated venous access. The rate of complications associated with filter retrievals that required advanced technique was significantly higher than seen with routine technique (5.3% vs 0.4%). Longer dwell time, more transverse tilt, and the presence of an embedded hook were associated with significantly increased rates of failed retrieval via routine technique.	IVC filter retrieval can be achieved with a high success rate by using advanced techniques in cases in which routine techniques have failed. There is a high overall success rate (98.2%) and a low complication rate (1.7%). However, there is an increased risk associated with proceeding to the use of advanced retrieval techniques, which should be weighed against the benefit of filter retrieval on a patient-to-patient basis.
26	Kiefer et al., 2016 [27]	USA	Retrospective study (n=100)		For use of comparison, there were tip-embedded filters in 59/100 patients (59%). AP venography detected 31 tip-embedded filters (53% sensitivity) and two false positives (95% specificity). Rotation venography detected 58 tip-embedded filters (98% sensitivity) and one false positive (98% specificity). This provided an overall accuracy of 70% for AP venography and 98% for rotational venography in tip-embedded filter detection. A significant difference was found between the sensitivities of the two approaches (p<0.01).	Rotational venography can be a useful diagnostic approach for tip-embedded filter detection prior to IVC filter retrieval when compared to AP venography. This is useful prior to retrieval because it is a determinant factor in which approach will be taken.
27	Avgerinos et al., 2013 [28]	USA	Retrospective study (n=401)		Prophylaxis occurred in 67% of patients. 259 retrievals were attempted and 237 filters were successfully retrieved (retrieval rate = 59.1%). 11/259 (4.2%) attempts were abandoned due to significant thrombus within the filter and were unsuccessful. In 142 patients no attempt for filter retrieval was made due to physician oversight (44.3%). 38/248 (15.3%) filter retrievals were recorded as challenging. Failed retrievals were predicted by prolonged dwell time (96.9 ± 111.9 vs. 29.5 ± 25.1 days), therapeutic indication, and filter wall apposition. Challenging retrievals were predicted by dwell time (51.1 ± 69.8 vs. 29.1 ± 24.5 days), filter tilt, and filter wall apposition.	Physician oversight leads to poor IVC filter retrieval rates. Retrievals can be challenging or fail when the dwell time is greater than 50 days and greater than 90 days, respectively, and when the filter hook apposes to the caval wall. Filter tilt can increase retrieval difficulty but does not affect failure rates.
28	Lee et al., 2015 [29]	USA	Retrospective study (n= 628)		Filters were removed successfully in 576/ 628 patients (92%). The mean IVC filter dwell time for successful removal was 85 days and the unsuccessful retrieval attempt's mean dwell time was 145 days (p=0.001). Retrieval success rates were similar for the common filter types, with notably lower retrieval rates for the GTF (89%) when compared to ALN (93%), Celect (93%), and Optease (91%).	The study recognized limitations due to only obtaining data at the time of retrieval. Overall, the study provides insight into optimal filter dwelling time for successful retrieval in differing filters.
29	Gotra et al., 2018 [30]	Canada	Retrospective study (n=447)		488 IVC filter retrieval attempts, 94.1% were successful. The ALN filter had the highest mean absolute value of tilt (5.6 degrees), the Optease filter had the largest mean migration (−8.0 mm) and the Bard G2 filter had the highest mean penetration (5.2 mm). The dwell time of 0-90 days or 90-180 days, net tilt of 10-15 degrees, caudal migration of -10 to 0 mm, and penetration less than 3 mm were positive predictors of successful retrievability. Higher odds of successful retrieval were obtained for the Bard G2X, Bard G2, and Cook Celect compared to the ALN and Cordis Optease filters.	Shorter dwell time, lower mean tilt, caudal migration, and less caval wall penetration are positive predictors of successful IVC filter retrieval.
30	Ramaswamy et al., 2018 [31]	USA	Retrospective study (n= 189)		Retrieval failure rates recorded were 11.6% for Option, 5.1% for Tulip, and .9% for Denali filters (Denali vs. Option p=0.018; Denali vs. Tulip p=0.159; Tulip vs. Option p=0.045). Filter retrieval failures were notably higher for the Option filter in comparison to the Denali and Tulip filters. Median fluoroscopy time for filter retrieval was highest for Option filter at 6.75 min, 4.95 min for Tulip, and lowest for Denali at 3.2 min (Denali vs. Option p<0.01; Denali vs. Tulip p<0.01; Tulip vs. Option p=0.67). Advanced retrieval techniques were used in 21.2% of Option filters, 10.8% of Tulip filters, and 0.9% of Denali filters (Denali vs. Option p<0.01; Denali vs. Tulip p<0.01; Tulip vs. Option p<0.01). The Denali filter required the least amount of fluoroscopy time and advanced technique used compared to both Option and Tulip filters.	The study concluded that the choice of the Denali filter and Tulip filter would be of benefit to a patient compared to the Option filter because of the significant findings during retrieval. The findings showed that despite there being many similarities in each of the filters, the Options filter had the highest retrieval failure rate, radiation time, and use of advanced techniques.
31	Laidlaw et al., 2021 [32]	USA	Retrospective study (n=252)		The mean IVC diameter at placement was 19.2 ± 3.3 mm. Mean filter tilts at placement and retrieval were 6.1 ± 4.9◦ and 5.2 ± 5.0◦, respectively. The mean tilt change was 5.0 ± 5.0◦. A larger IVC diameter was associated with greater filter tilt change. Greater tilt change and prolonged dwell time were associated with increased advanced retrieval technique use. Results were unchanged in a subgroup analysis of patients treated with GTFs.	Larger IVC diameter predicts increased filter tilt change, associated with challenging retrievals. Increased attention to IVC diameter during filter placement can address tilt-related complications.
32	Dinglasan et al., 2012 [33]	USA	Retrospective study (n=15)		Between January 2002 and December 2010, 148 IVC filters were retrieved, 15/148 filter bodies were fractured, and all were successfully retrieved. Nine of 15 fractured filters (60%) were removed in their entirety by using endobronchial forceps to retrieve the filter body and/or fractured struts. In three cases, forceps were used to retrieve the filter body, and the fractured strut was removed with a snare. In 6 patients (40%), only the filter body could be removed, three with the Recovery Cone and 3 with endobronchial forceps. Three failed attempts to remove fractured struts were made, with no attempt made in the remaining three. These struts were incorporated in the right ventricle, embedded in the IVC wall, or extraluminal. Minor caval defect was identified in 5/15 retrievals (33%); mild hemoptysis was noted in one case where the strut was snared from a pulmonary artery. No major complications occurred.	Fractured IVC filter bodies can be safely removed. Fractured filter struts can be removed when accessible, but are very often in a position that makes retrieval not possible.
33	Morrow et al., 2020 [34]	USA	Retrospective study (n=294)		295 filter retrieval attempts in 294 patients. No procedural IVC ruptures, morbidity, or mortality occurred. Retrieval was successful for 249 filters (84.4%). The median filter dwell time was 196 days for the successful retrievals and 375 days for the failed retrieval attempts. Penetration of the filter tines through the caval wall occurred in 291 filters (98.6%). However, the hook/apex (HA) of 31 filters (10.5%) had become embedded or had penetrated through the caval wall. The hook/apex and collar (HAþC) of 33 filters (11.2%) were embedded or had penetrated through the caval wall. The failure rate of filter retrieval with the HA embedded was 48.4% (15/31). The failure rate with the HAþC embedded was 66.7% (22/33). The failure rate for filters without these issues was 3.9% (9/231). The failure rate for HA and HAþC was greater than that for those without these issues but did not differ between the two issues. CT scans showed that the association of the filter with other adjacent retroperitoneal structures did not result in an increased rate of retrieval failure. Complex retrieval methods involving endobronchial forceps, ballooning, or snaring the collar of the filter were associated with increased retrieval failure compared with simple retrieval involving snaring the hook of the filter. The failure rates decreased over time. 8% of Patients with failed retrieval attempts experienced subsequent venous thromboembolism.	IVC filter retrievals are considered procedurally safe. Tine penetration was universal and did not significantly affect retrieval rate failure; however, IVC filters with the HA or HAþC embedded into or penetrating through the caval wall were a predictor of retrieval failure.
34	Puller et al., 2021 [35]	USA	Retrospective study (n=540)		77 patients had retained IVC filter fragment(s) after complex filter removal. 37/77 patients (14 men, 23 women) had adequate imaging follow-up to assess positional stability of the retained fragments, the remainder were excluded from further analysis. Excluding fractured foot processes, 51 separate filter fragments were retrospectively identified and followed for a median duration of 726 days. Filter designs producing the studied fragments included Celect, G2, Recovery, Günther, OptEase, Meridian, and G2X/Eclipse. 50/51 (98%) fragments were found to be unchanged in position during their respective intervals of observation.1 fragment displayed a rotational change without migration from its original location. No further fragment fractures or clinical sequelae were observed among the group.	A specific subset of patients who undergo IVC filter removal will retain extravascular filter fragments that were produced prior to filter manipulation. If asymptomatic, retained IVC filter fragments are mostly stable and can be safely followed on an intermediate-term basis
35	Lee et al., 2018 [36]	USA	Retrospective study (n=556)		Outcome and CT/Cavography were available from 335 patients, with a median CT follow-up time of 45 days (average: 126 days) and a median cavography follow-up time of 90 days (average: 102 days). IVCF leg perforation of the inferior vena cava wall greater than 3 mm was identified in 65 cases (19.4%) on follow-up imaging (64 cases). In addition, filter tilt greater than 15 degrees was identified in four filters (1.2%), filter migration greater than 2 cm in one filter (0.3%), and occlusive filter or IVC or iliac vein thrombus in 11 filters; no filter fracture was observed. One case of pulmonary embolism and two indeterminate cases were identified on follow-up CT pulmonary angiography (2.6%-7.7%). Retrieval was successful in 155 of 155 patients (median indwell time, 90 days; range, 1-445 days); an advanced retrieval technique was used in 11 retrievals	No fractures, a low migration rate (0.3%), a high retrieval rate (100%), and a total incidence of asymptomatic IVC perforation greater than 3 mm of 19.4% based on CT and cavography follow-up (28.6% for patients with CT follow-up only). Similar findings to the first generation select filter.
36	Lee et al., 2014 [37]	South Korea	Retrospective study (n=45)		IVC penetration following filter placement occurred in 87.6% of patients, and 57.8% of those involved significant penetration. Embedding of filter tips (i.e. lateral tilting) was observed in 51.1%. Asymptomatic vertebral body erosions and aortic penetrations were seen in 4.4%. Longer indwelling duration of the IVC filter was significantly associated with a higher grade of IVC penetration, and the risk of significant IVC penetration increased in patients with a filter indwelling time of more than 20 days and an IVC diameter of less than 24.2 mm.	IVC penetration on CT was common, and significant IVC penetration was associated with a longer indwelling time of the retrievable IVC filter and a lesser IVC diameter.
37	Kuo et al., 2013 [38]	USA	Prospective study (n= 50)		The IVC retrieval was successful in all of the 50 cases where the mean implantation period was 815 days with filters such as G2X, G2, Eclipse, Recovery, ALN, Celect, OptEase, and Simon Nitinol. The vascular remodeling such as neointimal hyperplasia and fibrosis was observed in 46 of 48 specimens with 96% adherent tissues. 1,082 days was the mean indwell time for fractured filters (n=31); however, the indwell time for the non-fractured filters (n=19) was 408 days. 61 conical IVC fractured filter components were found to penetrate intravascularly (n=26) and extravascularly (n=35). Of the 25 fractured components that penetrated intravascularly, 11 of them were free-floated in IVC, and 15 of them were embolized centrally. The electron microscopy studies showed that the mode of the IVC filter fracture was high-cycle fatigue (n=53), overload (n=6), and indeterminate (n=2). Upon retrieval of the filter, the lifelong anticoagulation prescription was ceased in about 97% of patients. Moreover, all the filter-related symptoms were alleviated in all 26 cases such as IVC occlusion, occlusion, component embolization, penetration-induced abdominal pain, and duodenal injury. Upon the mean follow-up of 371 days, no long-term complications were observed in patients.	The study concluded that the risk of the filter fracture increases after the in-dwell time of 408 days of its implantation and shows extravascular penetration and intravascular embolization symptoms. In addition, patients with failure of standard filter removal techniques were able to alleviate the filter-related morbidity and cease the use of anticoagulation by safely removing the filters with complex methods.
38	Tullius Jr. et al., 2016 [39]	USA	Retrospective study (n= 333)		The CT imaging results showed that the specific locations showed symptomatic indications of 38.3% for the filters placed <1 cm below the lowest renal vein, 27.1% for filters placed >2 cm, and of 27.1% for filters placed 1-2 cm below the renal vein. Further, the study showed significant differences in caval struct penetration, penetration of adjacent viscera, time to penetration, filter migration, or tilt when the filter was placed in infrarenal IVC. None of the filters were found to be fractured. In addition, none of the complex filter retrievals showed any incidence of patients with pulmonary embolism, and only one filter retrieval failed.	The study concluded that the filter apex location within infrarenal IVC placement of >2 cm below the level of renal vein inflow is not related to the difference in indwelling or retrieval complications. It was recommended to determine the filter located in the region of IVC based on its size in order to optimize the vertical axis of the filter and to avoid the tile in cases where the IVC is at an angle.
39	Ganga et al., 2021 [40]	India	Retrospective study (n=31)		IVC filters were placed in 50 patients, and data was retrieved for 31 patients (mean: 51.24 years, 67.74% males). According to ACCP/AHA guidelines, 24 (77.42%) had an absolute indication for IVC filter. All 31 IVC filters were temporary, 23 (74.19%) of which were placed via femoral access. 29 (93.55%) patients had infrarenal IVC filter placement. The average tilt at deployment was 3.71, whereas it was 5.3 at retrieval. There were no periprocedural complications or filter migrations during placement or retrieval. Retrieval was attempted in 11 (35.48%) patients and was successful in 10. The mean indwelling time in this group was 158.55 days (range: 55-366 days).	Study reveals low IVC filter implantation rates which are predominantly for absolute rather than relative indications. Poor retrieval rates reflect the urgent need for better patient and physician awareness. Periodic follow-up is needed to improve the IVC filter retrieval rate and to prevent complication rates.
40	Brahmandam et al., 2019 [41]	USA	Retrospective study (n=191)		There were 191 IVC filter retrievals (SR: 157; AER: 34) in 183 patients (mean age: 55 years; 51% male). 15 filters (7.9%) were placed at an outside hospital. The indications for placement were mostly therapeutic (76% vs 24% for prophylaxis). All IVC filters were retrievable. Venous ultrasound examination of the lower extremities of 133 patients (70%) was performed before retrieval, whereas only five patients (2.6%) received a computed tomography scan of the abdomen. There was no difference in the mean filter dwell time in the two groups (SR: 147.9 ± 146.1 days; AER: 161.4 ± 91.3 days). AERs were more likely to have had prior attempts at retrieval (23.5%) compared with SRs (1.9%). The most common AER techniques used were the wire loop and snare sling (47.1%) and the stiff wire displacement (44.1%). Bronchoscopy forceps were used in four cases (11.8%); this was the only off-label device used. AERs were more likely to require more than one venous access site for the retrieval procedure (23.5% vs 0%). AERs were significantly more likely to have longer fluoroscopy time (34.4 ± 18.3 vs 8.1 ± 7.9 minutes) and longer total procedural time (102.8 ± 59.9 vs 41.1 ± 25.0 minutes) compared with SRs. The complication rate was higher with AER (20.6%) than with SR (5.2%). Most complications were abnormal radiologic findings that did not require additional intervention. The procedural cost of AER was significantly higher (AER: $14,565 ± $6,354; SR, $7,644 ± $2,810; P<0.001) than that of SR. This translated to an average increase in the cost of $6,921 ± $3,544 per retrieval procedure for AER.	Advanced endovascular techniques provide a feasible alternative when standard IVC filter retrieval techniques do not succeed. However, these procedures come with a higher cost and higher rate of complications.
41	Turba et al., 2009 [42]	USA	Retrospective Study (n= 92)		GT filters were accessed via the right internal jugular vein (n=49), right femoral vein (n=37), left femoral vein (n=4), and unknown (n=2). 83.7% of the filters were located infrarenal, with 5.4% located suprarenal. Only 10% (n=9) of patients had a small thrombus attached to the filter and 90% had no thrombus related to the filter. IVC filter tilt degree at the time of placement was between 0-19 degrees (mean 6.6 degrees). At filter retrieval, the tilt degree ranged between 0 and 19 degrees (mean 6.47 degrees). In 15% of patients, the filter hook was abutting the IVC wall. Complete endothelization of the filter hook was seen in 11% of patients. One or more of the filter primary struts were buried in the IVC to the level of secondary filter struts in 54% (n=50) of patients. An initial attempt to retrieve the filter was made with a loop snare conventional technique with a success rate of 88% (n=81). The MTGL technique was performed in patients with buried filter hooks (n=8) after the failed conventional technique. Two cases had buried filter hooks, but the surgeons did not use MTGL based on preference. MTGL was successful in six patients, and not in two. Overall successful filter retrieval went from 88% to 95% with the employment of MTGL. Unsuccessful retrievals were at 5% (n=5).	The study focused on GT filter dwell time, filter strut penetration/endothelization, and IVC wall and hook relationship. The study concluded that the use of alternative techniques maximizes filter retrieval success rate.
42	Tamrazi et al., 2015 [43]	USA	Retrospective study (n= 12)		Successful removal of IVC filters was performed in 11 of 12 patients (91.6%). In the remaining patient, a chronically thrombosed IVC filter could not be removed even with the use of laser sheath assistance. It was recanalized with PowerWire RF guidewire and a stent was placed. There was the occurrence of a major complication described as a large venous groin hematoma.	The study found that percutaneous IVC filter removal can be performed in specific patients, even with longer dwelling times. The removal of chronic IVC filters can be accessed through jugular and femoral approaches in conjunction with laser sheath assistance. Increased risk of failure was associated with chronic filter thrombosis and tissue scarring.
43	Anzai 2013 [44]	Japan	Questionnaire-based Study (n=11)		The average IVC filter dwelling time was 816 days (range: 32-2,776 days). The success rate of removal of GT retrievable IVC filters was 64% (7 of 11). The success rate was significant in relation to shorter dwelling times (112 ± 143 days vs. 2,047 ± 558 days; p<0.001). The longest dwelling time that resulted in successful IVC filter removal was 433 days.	The study found IVC filter dwelling time to be significantly associated with removal success. The study also notes that patients with IVC filters of less than a year have sufficiently higher chances of successful IVC filter removal.
44	Sangwaiya et al., 2009 [45]	USA	Clinical study (n=73)		14 out of 15 filters were retrieved successfully after a median period of 84 days. All filters were successfully deployed in 61 patients. Four filters (6.5%) showed significant tilt. After the meantime period of 62 days, three patients developed PE due to filter placement. However, further imaging showed only 2 of 3 patients showed new PE. No filter migration was reported in follow-up imaging with radiography (n=32), CT (n=11), and angiography (n=4) in 47 patients after a mean period of 62 days. Abdominal CT showed filter-related problems in seven patients (39%) including penetration of filter legs in four patients and fracture/migration of filter was found in one of the patients resulting in a thrombus in the IVC.	The caval filter leg penetration is one of the complications of using Celect IVC filters, which is related to high incidence rates. Upon the filter placement safely, the symptomatic PE after the filter placement was observed in 2.8% of patients via CT scan.
45	Stavropoulos et al., 2014 [46]	USA	Prospective study (n=200)		The nitinol IVC filters showed remarkable results where the clinical success of filter placement was achieved at 94.5% of patients and the technical success rate was 99.5%. In addition, the filter retrieval technical success rate was 97.3% where 108 filters were retrieved from 111 attempts made. The two of these cases were not successful due to the inability to engage the filter apex with a snare and in one case the IVC filter was engaged but could not be removed. The mean dwell time of the filter retrieval was 165 days with no complications related to filter fracture, migration, or tilt greater than 15 degrees during the time of retrieval of the six-month follow-up.	The study concluded that the nitinol retrievable IVC filter is safe can be used to provide protection against pulmonary embolism and could be retrieved with a low rate of complications.
46	Stevens et al., 2023 [47]	Australia	Retrospective study (n=453)		This study followed 453 patients, with 272 individuals in the pre-MDST and 181 individuals in the post-MDST. The study found that compared with the post-MDST (73.5%) patients, pre-MDST (52.6%) had lower IVC filter retrieval rates.	The study concluded that although the MDST team was associated with higher retrieval rates and shorter IVC dwell time, there was no clinically significant difference found in the rate of complications for patients.
47	Guzman et al., 2016 [48]	USA	Retrospective study (n= 20)		The success rate for IVC filter retrieval was 100%. Filter indications included contraindication to anticoagulation, free-floating thrombus, post-trauma pulmonary embolism prophylaxis, and pre-thrombolysis pulmonary patient. The standard retrieval technique was used in 17/20 of the patients (85%). Standard retrieval with adjunctive techniques was performed in 3/20 patients (15%). These advanced techniques included double-snare technology, balloon assistance, and endobronchial forceps retrieval. Two retrieval-related complications occurred: IVC mural dissection noted on venography and snare catheter fracture requiring removal.	IVC filter retrieval can safely be done in children, but there are chances it will be challenging in cases of filter tilt or embedding. The study concluded that in cases of complicated retrieval, using adjunctive techniques can increase filter retrieval rates when the standard technique is not enough.
48	Moriarty et al., 2020 [49]	Australia	Retrospective study (n=23)		The study reviewed procedures from 2012 to 2019 that necessitated a "low-profile" hangman technique. In 473 filter retrieval attempts, 66 utilized advanced techniques with 23/66 procedures using specifically the hangman technique. 22 of the 23 procedures were successful on the initial attempt. No procedure-related complications were found.	The study reviewed the low-profile hangman technique being utilized in the case of complicated IVC filter removals, specifically embedded hooks. They found that using 11 French venous access sheaths was effective, as well as low cost for complex filter retrieval.
49	Ahmed et al., 2020 [50]	USA	Retrospective study (n=378)		A total of 462 filter retrieval attempts were made. Success rates for standard and advanced retrieval attempts were 86.8% (317/365) and 91.8% (89/97), respectively. The rate of periprocedural complications was significantly high in the advanced retrieval group. Complication rates for standard and advanced retrievals were 0.6% (2/318; minor) and 5.2% (5/97; 3 minor (3.1%) and 2 major (2.1%)), respectively. The two major complications during advanced retrievals were filter fracture and embolization. Average fluoroscopy time for advanced retrievals was significantly higher than for standard retrievals (23.1 min vs 4.3 min). The average radiation dose for advanced retrievals was also significantly higher than for standard retrievals (557.2 mGy vs 156.9 mGy). The use of general anesthesia was significantly more prevalent in advanced retrievals compared to standard retrievals (6.2% vs 0.9%).	Advanced filter retrieval and standard snare retrieval results are similar since both experienced high rates of success, but advanced filter retrieval is associated with greater fluoroscopy time, anesthesia requirements, and radiation exposure.
50	Bikdeli et al., 2017 [51]	USA	Randomized control trials (n=6); prospective observational studies (n=5)		Patients receiving IVC filters had a lower risk for subsequent pulmonary embolism (OR=0.5, 95% CI (0.33 to 0.75)), but showed an increased risk of DVT (OR=1.70, 95% CI (1.17 to 2.48)), non-significantly lower PE-related mortality (OR=0.51; 95% CI (0.25 to 1.05)), and no change in all-cause mortality (OR=0.91; 95% CI (0.70 to 1.19)).	The study concluded that the use of IVC filters resulted in a reduced risk of subsequent PE, increased risk of DVT, and nonsignificantly lower PE-related mortality. However, the study showed that there is no difference in all-cause mortality.
51	Cho et al., 2015 [52]	South Korea	Retrospective study (n=25)		16 out of 25 (64%) filters showed a range of complications such as IVC wall penetration (n=11, 44%), tilted within IVC (n=6, 24%), embedded struts (n=3, 12%), and fracture of the strut (n=3, 4%). Of these patients, five of them showed overlapping complications. Two of them (8%) had also complained of filter-related pain. The success rate of IVC filter retrieval by double-loop technique was 14/16 (87.5%). There were no major filter retrieval-related complications.	The study concluded that the complex procedure of the double-loop technique is safe and a feasible method for complicated IVC filter retrieval.
52	Holly et al., 2018 [53]	USA	Retrospective study (n=17)		Retrieval success for 17 patients, who had at least one strut in either the aorta or iliac artery, was 100% without any complications. Follow-up from 10 days to two years was available for 12/17 (71%) patients, and no delayed complications were reported. The retrieval techniques included standard snare retrieval in three patients, loop wire technique in eight cases, rigid endobronchial forceps in five cases, and rigid endobronchial forceps with excimer laser sheath-assisted photothermal ablation in one case.	The study reports that long-dwelling IVC filters with aortoiliac strut penetration can be successfully and safely retrieved. They advised that given the small sample, an experienced operator with resources such as stent grafts and occlusion balloons to be used in preparation for sudden complications.
53	Yamagami et al., 2009 [54]	Japan	Retrospective study (n=76)		Mean implantation or dwell time for the IVC filters in the 76 patients was 19.7 ± 28.6 (SD) days. The mean follow-up after filter retrieval was 39.8 ± 22.1 months. In 5 of 76 patients (6.6%), worsened VTE was seen after filter retrieval. Two patients (4.0%) had worsened or recurrence of DVT in their lower extremities, two patients (2.6%) had developed PE, and one patient had a second IVC filter placed permanently. The mortality rate during the follow-up period was 2.6% (n=2), both due to PE with no potential risk factor for VTE.	Important conclusions from the study included a note of the two patient deaths due to recurrent PE, when they revealed no particular risk factors except for anticoagulant interruption in one of the cases. The study suggests caution to be employed when regarding IVC filter retrieval indication and clinical follow-up after removal. The study emphasizes the need for larger studies to better determine indications for IVC filter removal.
54	Ho et al., 2015 [55]	Australia	Retrospective Study (n=223)		36 patients (16%) developed DVT or VTE subsequent to placement of IVC filters. 27 had lower limb DVT, 8 upper limb DVT, and 4 pulmonary embolisms. A high Injury Severity Score, tibial/fibular fractures, and a delay in initiating pharmacological thromboprophylaxis after insertion of the filters (14 vs. 7 days) were significant risk factors. 30 patients were lost to follow-up (13%) and their filters were not retrieved. Mechanical complications including filters adherent to the wall of IVC (4.9%), IVC thrombus (4.0%), and displaced or tilted filters (2.2%) were common when the filters were left in situ for more than 50 days.	Delays in initiating pharmacological thromboprophylaxis or filter removal were associated with an increased risk of DVT, VTE, and mechanical complications of retrievable IVC filters in patients after major trauma.
55	Swami et al., 2014 [56]	USA	Retrospective study (n= 254)		This study followed 254 vena cava filters which were placed for absolute venous thromboembolism (n=65), relative venous thromboembolism (n=28), and lastly for prophylaxis of venous thromboembolism (n=161). Upon follow-up after 108.5 days with the imaging study of 96 patients, 15 of them showed complications such as filter migration, and penetration of IVC filter. The successful retrieval of 19 IVC filters was observed.	The study concluded that the strict protocols for the follow-up after IVC placement need to be placed for the timely retrieval of the filter and most of the filters that were placed for prophylaxis reasons showed a low retrieval rate.

IVC filters are invaluable in the prevention of PEs in patients who are refractory or contraindicated to anticoagulation, our aim was to identify the factors that contribute to complications associated with IVC filter retrievals. Many studies have shown a strong association between IVC filter dwell times and IVCF retrieval complications. A study by Averginos et al. was included, which showed the significance of prolonged IVCF implantation time as a strong predictor of failed or challenging retrievals [28]. 

The striking outcome result of our research search showed that many well-referenced complications of IVCF retrieval can actually be an outcome of prolonged filter dwell times. In support of this, two studies showed that IVCF fracture rates increased as dwell durations lengthened [16,37]. Other studies have documented how the increased IVCF implantation time has been correlated with IVC wall penetration, perforation, and filter migration/tilting. All of these complications lead to difficulty during standard retrievals, which lead to increased failures or the necessity for stronger force/complicated procedures. 

Complicated retrieval procedures used for IVC filters that have migrated, tilted, or penetrated IVC walls include but are not limited to balloon-assisted, wire loop-and-snare, irregular bronchoscopy, and laser excimer sheath. These procedures have been documented to have led to filter leg fragment embolization to the pulmonary artery, as well as resulting IVC pseudoaneurysms, arteriovenous fistulas, and wall perforation and subsequent internal bleeding [14,26,49,51,52]. These procedures are also associated with longer surgical time, and patients are exposed to a longer duration of general anesthesia, which can be a significant detriment to these patients [50]. 

Although standard retrievals are overall preferred due to the decreased complication rates, during the comparison of complex techniques, rigid endobronchial forceps techniques were found to be much more effective in retrievals than the balloon-assisted method or loop-and-snare techniques [52]. Interestingly, studies found that using a thicker sheath of 12F is much better in retrieving the filter than the classically used 8.5F sheath due to its inability to completely encircle the filter. This technique was beneficial for the retrieval of conical, Gunther-Tulip, and Celect filters [52,53]. 

As discussed, filter position and minimized dwell times are crucial in the safety of IVC filter retrieval and different brands of filters have shown significant variations specifically associated with IVCF migrations/wall penetration and complication rates. The ALN filter was associated with a greater degree of filter inclination in comparison to Denali filters [2]. Importantly, as we have documented dwell times to contribute to complications, the conical option and the Gunther-Tulip filter (GTF) exhibited the least complications after increased dwell times [1].

Overall, reduced dwell time was found to be beneficial in decreasing the number of complications associated with filter retrieval. Morrow et al. found 196 day median dwell time to be associated with successful retrievals [34]. Studies have shown effectiveness in minimizing dwell times through proper follow-up due to successful patient education or the implementation of multidisciplinary surveillance teams (MDST). MDST cohorts had a significant increase in successful retrievals compared to the cohort that did not have an MDST implemented, as well as significant decreases in filter median dwell times [47]. 

Kuo et al. highlighted an important post-retrieval complication of acute partial caval thrombosis associated with patients who did not receive anticoagulation treatment prior to or during retrieval. The application of anticoagulation prior to/during is strongly recommended, one study showed that the complication was completely eliminated after the addition of anticoagulation [12]. Anticoagulated patients have decreased occurrences of dislodged emboli during retrieval as well as decreased risk of new-found thrombosis [52,55,56]. 

Discussion

Complications associated with IVC filters were found to be influenced by a variety of factors, including filter positioning, filter dwell time, retrieval technique, and coagulation. Compared to permanent filters, retrievable IVC filters are safer and more effective at preventing the recurrence of pulmonary thromboembolism (PTE), according to the literature [18]. Furthermore, temporary filters exhibited a reduced mortality rate (16%) compared to permanent filters (35%), immediately following implantation. PTE recurrence was observed in 18% of patients who underwent filter removal; conversely, retrievable filter recipients did not exhibit PTE [18].

Filter retrieval difficulties are frequently observed in patients who are contraindicated for anticoagulants, have a history of intracranial hemorrhages, increased rates of immobility, or malignancy [6,19]. Filter embedment is a likely second most frequent complication, after thrombosis formation at the filter site [20].

To prevent these potentially fatal complications in patients who have chronic retrievable IVC filters, it is strongly advised that they maintain ongoing care, have an electronic communication follow-up method with their healthcare providers, and have the filters removed when clinically appropriate [21-24]. Research has indicated that the probability of successful IVC filter removal rates is substantially impacted by whether or not the removal is scheduled during the placement encounter [25].

Filter Position

Embedded hooks, caval wall penetration, and filter malpositioning (tilts) are frequently linked to the requirement for sophisticated or intricate methodologies [10,26]. According to one study, the location and position of IVC filter limb penetration could be determined using CT scans performed prior to retrieval procedures; 89% of those filters were subsequently retrieved successfully [10]. By accurately identifying 58 out of 59 tip-embedded filters, rotational venography demonstrated its efficacy in reducing the complexities associated with retrieval methods that are complex in nature [14,27].

An additional prevalent complication observed was IVC filter inclination and angulation surpassing 15 degrees, which was linked to unsuccessful and difficult retrievals [28]. ALN and Optease filters had the highest rate of IVC filter retrieval failure, according to Gotra et al. [29,30]. This was because the ALN filter had the greatest mean absolute value of tilt, and the Optease filter had the greatest mean migration. In comparison to Denali filters, Option IVC filters were associated with a greater degree of filter inclination change, a greater demand for sophisticated retrieval techniques, and a higher failure rate [14,31,32].

Filter struts penetrating the right ventricle, IVC wall, extraluminal tissue adjacent to the IVC, filter fragment dislodgement, and concomitant aortic/vertebral penetration were cited as reasons for failed retrieval attempts in the reports [18,33]. Greater retrieval failure rates are associated with the lateral angling and embedding of the hook/apex or collar of the filter compared to filters lacking penetration [33,34]. Although the majority of research indicates that filter position is crucial for IVC filter retrieval complications, such as dislodgement of IVC filter fragments, Puller et al. propose that in the case of asymptomatic patients, filter fragments are generally stable and can be safely monitored [35]. In general, the safety of IVC filter retrieval is enhanced in the absence of migrations, penetrations, or tilts [36].

Dwell Time

Extended dwell time is associated with enhanced advanced retrieval techniques, according to Laidlaw et al. [32]. Certain studies have documented a potential for significant complications during the procedure due to the use of excessive force in retracting the filter [14,23]. As stated by Quencer et al., the misalignment of the retrievable IVC filters results in an extended filter retention time, which in turn contributes to the prevalent issues of filter inclination and IVC wall perforation [14]. It is estimated that conventional methods are incapable of extracting 40-60% of retrievable IVC filters that have been implanted for more than a year [26]. According to the studies, fifty percent of filters that were implanted for more than two and a half million years but were not removed after twenty years did not undergo retrieval using standard techniques or failed follow-up [23]. More than 1.1 million patients in the United States would receive embedded filters regardless of whether the filter is indicated [23].

The median filter dwell time for successful retrievals in Morrow et al. was 196 days, while it was 375 days for unsuccessful retrieval attempts [34]. Prolonged dwell time was found to be a predictor of unsuccessful retrievals (96.9 ± 111.9 days) and challenging retrievals (51.1 ± 69.8 days), according to Averginos et al. [28]. A greater degree of IVC penetration was significantly correlated with a prolonged indwelling duration of the IVC filter. Patients with filters in place for longer than 20 days had an elevated risk of significant IVC penetration [37]. Additionally, IVC filter fracture rates increase as dwell durations lengthen [16,37]. In support of this claim, Kuo et al. provide evidence that the average indwell duration of fractured filters was considerably longer at 1,082 days compared to 408 days for non-fractured filters [38]. Additionally, a reduced dwell time was found to be a positive predictor of successful IVC filter retrieval [30]. The results of this research indicate that studies that failed to consider variations in dwell durations might have exaggerated the rates of successful IVC retrieval [36,39-41].

According to Al-Hakim et al., the average filter dwell time in their study is 134 days (with a range of 0-2,475 days) [26]. Furthermore, they find that extended dwell times are substantially correlated with higher rates of unsuccessful retrieval using routine techniques. A growing filter dwell duration is correlated with more complex retrieval techniques and lower retrieval success rates, according to research [10,13,42]. This is because of the increased risk of strut, tilt, and caval wall penetration. Nevertheless, a 2013 study by Pellerin et al. demonstrated that an extended implantation duration does not inherently correlate with a heightened risk of filter extraction failure. Despite the detection of filter inclination and IVC penetration, none of the ALN filter retrievals experienced failure or complications during the extraction process following a dwell time of over one year [2]. Approximately 25.6 months passed on average between implantation and retrieval, and every extraction was fruitful [2]. Additionally, Ileascu et al. discovered a recent account in which a Lynch filter was effectively eliminated 3006 days later [10]. An investigation pertaining to the percutaneous retrieval of permanent IVC filters, including Simon Nitinol and TrapEase, revealed an 8.4% rate of filter complication removal over a mean of 5.1 years (ranging from seven days to 15 years) [42,43]. This research demonstrated that retrieval of IVC filters was effective, as only two instances exhibited complications despite the extended residence periods of these permanent filters.

A complicated retrieval rate of 8% was documented by Given et al. in 2008, pertaining to GTFs, following an average implantation period of 76.40 days [7]. The longest retrieval occurred 309 days later. The extended dwell time and subsequent successful retrieval were corroborated by the GTF, which exceeded the manufacturer's initial suggestion of retrieval occurring no later than 14 days following filter insertion [7]. The absence of complications during retrieval follow-up for the GTF was documented in the studies conducted by Anzai et al. (2011) and Kuo et al. (2013), with retrieval mean dwell times of 304 and 433 days, respectively. Furthermore, a correlation between the duration of filter implantation and extraction success rates was indicated in both investigations [8,44]. Recent investigations on the GTF, on the other hand, have produced results that contradict the aforementioned conclusions. Statistical analysis revealed a significant relationship between filter strut penetration and extended dwell periods. There is a negative correlation between the duration of dwell time and the efficacy of filter retrieval. This is attributed to the heightened likelihood of tissue embedding around the filter, which can result in dense fibrosis and neointimal hyperplasia [14,42,43].

Like the GTF, the conical Option IVC filter exhibited minimal complication rates during retrieval (7.7%) following an average dwell time of 67.1 days following filter implantation [1]. There were no fractures or embolizations caused by the filter. It was determined that none of the fatalities or DVTs were attributable to the filter, but rather to concurrent or preexisting conditions. Thus, 12% of the subjects exhibited clinical complications, as determined by the Options filter [1].

More than 150 instances of filter inclination associated with the risk of new PE and recurrent PE with a caval filter have been reported for the conical Celect filter [7]. The study also found that Celect filters had a comparatively low convoluted retrieval rate of 7%, in contrast to the GTF's 8% [7]. Following an average of 121 days of residence time and a design modification derived from the GTF, these outcomes were exceptional. In comparison to GTF, the Celect IVC filter demonstrated safety during deployment and retrieval; however, in one instance, removal was unsuccessful as a result of a thrombus in the IVC. The literature extensively covers the high incidence of IVC wall penetration of less than 3 mm by the filter legs or struts verifying PE after filter implantation (2.8% for both the G2 and conical Celect filters) [45].

The investigations conducted by Stavropoulos et al. regarding the Denali (Nitinol) IVC filter revealed that after 165 days and 200.8 days of dwell time, complicated retrieval rates of 2.7% and 2.4% of the filter, respectively [45]. In both investigations, filter struts or filter hooks that are frequently encountered during filter removal and are embedded in the IVC were not observed [3,46]. Nevertheless, both investigations encountered two unsuccessful filter retrievals as a result of the anterior-posterior angulation of the IVC filter. It obstructed the clinician's ability to detach the filter catch using a snare. One drawback of the cited studies is that imaging is only performed on patients with symptomatic PE for the purpose of calculating the complication rate; patients with asymptomatic PEs who may not be diagnosed are also not accounted for [3,46].

After 122 days of residence time, the G2 filter study revealed no complications and successful retrieval on all twenty-seven attempts [22]. The study documented a mean filter tilt of merely 10° for the G2 conical filters. On the contrary, the venographic images of a single patient unveiled a gradual increase in filter inclination from 20° to 28°, with the apex of the filter aligned with the lateral caval wall. She was referred for filter retrieval 121 days after its insertion as the patient [22]. Therefore, a recovery cone system was necessary for this patient in order to facilitate apical cone snaring and effective filter retrieval through its reinforcement [22].

Following IVC filter placements, Stevens et al. observed 453 patients, of which 181 were followed after the MDST was established and 272 were in the pre-MDST cohort. The research discovered that the rate of IVC filter retrieval was greater in the post-MDST cohort (73.5% vs. 52.6% in the pre-MDST cohort) [47]. Additionally, the median latency time between IVC filter insertion and retrieval was reduced: 187 days for the pre-MDST cohort compared to 150 days for the post-MDST cohort [47]. An MDST team was correlated with a reduced length of stay and an improved prognosis for the patient.

Retrieval Technique

The retrieval of IVC filters is a straightforward process that requires only a coaxial sheath and a snare [14,41,48]. By administering the procedure jugularly, the standard retrieval device can easily latch onto the hook of the IVC filter, facilitating its removal. Filter mispositioning in the renal vein, filter tilt, encasement of the filter apex in the adjacent IVC wall, filter fracture, significant extracaval protrusion of filter elements, fibrin cap, or filter-associated thrombus are all potential causes of inability to engage the filter apex [49,50]. When such circumstances arise, sophisticated retrieval methods are implemented [10,13,14,48]. These methods consist of the suspension (wire loop-and-snare), endobronchial forceps dissection, the hangman technique, the utilization of laser excimers, balloon-assisted retrieval, and the double-snare technique. In advanced technique procedures, the incidence of complications during IVC retrieval escalated by a factor of four, from 5% to 10% for minor complications, and by a factor of 13 for significant complications, in comparison to the standard retrieval technique [14]. Advanced retrieval techniques are linked to several complications, including embolization, filter fracture, an increased average fluoroscopy duration, a higher average radiation dose, and a greater utilization of general anesthesia in comparison to standard retrievals [50].

Following the failure of the initial standard technique, the segregated "low-profile" hangman technique was implemented [14]. A standard 11 F Cook filter retrieval membrane is utilized for this method, which is more compact than alternative advanced removal techniques. Achieving a successful retrieval of the filter was a non-complication outcome for all 23 patients who participated in the study, both during and after the procedure. This apparatus is easily obtainable in the majority of interventional radiology practices, which confers benefits in terms of affordability and accessibility [26,49].

Irregular bronchoscopy A forceps retrieval is frequently mentioned as a sophisticated technique in cases where the IVC filter is ingrained within the caval wall. Among the most common complications is symptomatic IVC pseudoaneurysm, which necessitates balloon tamponade [14]. Two filter leg fractures necessitated embolization to the pulmonary artery and an asymptomatic IVC pseudoaneurysm constituted minor complications [14]. Trauma during the procedure has been identified as a drawback of this method, as evidenced by the identification of contrast extravasation in 8.3% of removals [14]. Reports of arterio-venous fistulas between the renal artery and IVC, as well as embolization caused by leg fractures, have been documented subsequent to retrieval [14].

The utilization of this technique is also necessary for embedded or tilted filters, in addition to the sling (wire loop-and-snare) method. In conjunction with leg fracturing, contrast extravasation, and unintended IVC dissection are frequently observed complications. It is possible that reorientation will be required throughout this procedure, particularly when handling malleable Nitinol filters [14]. Twenty consecutive patients experienced no complications as a consequence of the sling technique modification [14]. After aligning the filter, the hook was loop-snared. On the contrary, several further investigations carried out by Quencer produced contradictory results regarding this modified approach, resulting in an overall incidence of complications amounting to 20% [14].

In the process of filter retrieval, a laser excimer sheath is utilized when an embedded filter is situated above adhesive tissue. A study involving 251 patients, as documented by Quencer et al., indicated that this technique was linked to a 1.6% incidence of significant complications [14]. A venous pseudoaneurysm, contrast extravasation, and caval-enteric fistula accompanied by septic caval thrombophlebitis are a few potential complications [14]. Furthermore, 10% of patients experienced complications, including cholecystitis, renal infarction, and coagulopathic hemorrhages, according to Cho et al. In comparison to the conventional retrieval method, this technique produced exceptional outcomes for 414 distinct categories of retrievable filters and 86% permanent filters, with the latter exhibiting the longest dwell time of 8.5 years while requiring the least amount of force. It was discovered that this method produced an average of 1.8% complications and a retrieval success rate of 99.4% [14,23,51]. Therefore, this method aids in circumventing invasive open surgical procedures by safely extracting different IVC filters, irrespective of their dwell time [23].

Retrieving IVC filters that have penetrated the IVC wall requires the use of a number of complex techniques, including the balloon-assisted method and numerous loop techniques. Nevertheless, it is worth noting that the retrieval success rate for loop-snare and balloon-assisted techniques may be comparatively lower (80%) than that of rigid endobronchial forceps techniques [52]. A number of interventionalists regard endobronchial forceps as an aggressive technique [52]. The filter tip was rapidly secured using a modified snare-over-loop guide wire technique [52]. This involved inserting the Gooseneck snare into the 12 F sheath rather than the 8.5 F inner sheath. The complex IVC filters were completely encased in hypertrophic endothelial scar tissue, which posed a retrieval challenge when employing the standard 8.5 F sheath. In contrast to the 12 F sheath, the conventional 8.5 F sheath was unable to completely encircle the filter. The modified technique demonstrated an overall success rate of 87.5% in retrieving conical, Gunther-Tulip, and Celect filters. In contrast, non-conical Optease filters failed to retrieve in 33% of patients due to their greater contact area with the caval in comparison to conical filters [52,53].

Clotting

If the patient is taking anticoagulants and has a projected survival time of at least six months, the IVC filter can be safely removed, and there are no indications that the patient will require a filter in the future, then the filter may be removed [14]. Yamagami et al. determined that the mortality rate resulting from a PE subsequent to filter removal was deemed to be substantial at 2.6% [54]. The presence of this complication warrants prudence when deciding whether or not to remove an IVC filter. Consequently, by diligently and consistently monitoring patients at an outpatient clinic using D-Dimer assays and ultrasounds, the risk of PE post-retrieval was reduced to 2.0% [51,54]. Kuo et al. investigated the occurrence of acute partial caval thrombosis as a complication during a sequence of advanced IVC filter retrievals in all patients who did not receive anticoagulant treatment prior to or during surgery.

By administering anticoagulants to the remaining patients prior to and throughout the operation, this procedural complication was eliminated [12]. A novel device was developed because prophylactic heparin has not been shown to reduce the incidence of clinically significant PE, despite the fact that it has been the primary cause of mortality in critically ill trauma patients [3]. For these patients, the device integrates an IVC filter with a triple-lumen central venous catheter (CVC) for bedside administration [3]. This one-of-a-kind administration device permits the removal of the IVC filter prior to patient discharge. Complications linked to the removal of this device consist of the development or exacerbation of acute proximal lower extremity DVT. The occurrence rate of these incidents varied over time, with 7% transpiring on day seven and 18% on day 30 [3].

Hoppe et al. support the safety of IVC retrieval in anticoagulated patients. Nevertheless, reversing anticoagulant medications from warfarin to heparin during medical procedures raises the likelihood of venous thromboembolism (VTE) in patients [11]. Ho et al. observed that the insertion of IVC filters was associated with the development of DVT, VTE, and PE in the lower and upper extremities [55,56]. IVC filters do not effectively inhibit thrombus formation in patients who are non-compliant or have anticoagulant medications contraindicated. Anticoagulant-treated patients have a lower risk of entrapped emboli becoming dislodged during IVC filter removal than these individuals [56]. When contemplating the removal of IVC filters from these patients, extreme caution is required [52,55]. 

Limitations

Our review had several identifiable limitations beginning with the exclusion criteria outlined in the methods section, and specific search criteria used to identify articles pertinent to IVC filter removal/retrieval and complications. Additionally, our review did not contain a meta-analysis, to further strengthen our findings. Our search was limited to three databases (PubMed, ScienceDirect, ProQuest) and relevant papers may have been excluded. Our inclusion of only full-text and English articles may have limited us from relevant information and significant international findings. Our systematic review is limited by the quality/bias that may have been associated with each study reviewed, including selection bias for example. Retrospective cohort studies rely on the availability of information which may have been incomplete or limited by participant recall bias. Our prospective cohort studies are limited by the possible loss of follow-up. All cohort studies are vulnerable to confounding variables and publication bias, which could have interfered with the validity of the studies included. Despite the careful manner in which our systematic review was performed, we accept that our review may not contain all relevant studies and research relevant to our topic. 

Scope of future studies

Our review aimed to identify relationships associated with increased complication rates. We identified important variables such as filter position, dwell time, retrieval technique, and clotting and extracted associated outcomes from many different studies in order to find patterns. We hope that additional studies can further explore these relationships and expand on the factors contributing to increased complication rates. Future researchers can use our review as a foundation to build on the points established in this systematic review to further design and report additional studies. 

## Conclusions

IVC filter retrieval comes with many setbacks; our review suggests that the increased dwell time of the IVC filter is the main contributor to the complications encountered during retrievals. The retrieval technique of choice would be the standard technique, but often, chronic IVC filters lead to filter embedment, filter malpositioning, and increased thromboses at filter sites, necessitating the need for radically advanced techniques. Many factors can lead to complications during IVC filter retrieval, but timeliness is one thing that physicians can minimize. This is why we recommend proper patient education and communication and regular follow-up appointments, including IVC filter imaging or MDSTs, to ensure timely action. In conclusion, IVC filter retrieval complications can be mitigated by keeping patient relationships and thorough educational awareness. The low number of articles about IVC filter retrieval complications limited this study. Many of the articles included in our study also were based on small sample sizes of IVC filter removals.
